# Supplementary material for: Challenges Faced by Health Professionals in Obtaining Correct Medication Information in the Absence of a Shared Digital Medication List
Source: Pharmacy (Basel). 2021 Feb 22;9(1):46. doi: 10.3390/pharmacy9010046 (PMC8006028; doi:10.3390/pharmacy9010046)
Supplement: Supplementary file 1 [file pharmacy-09-00046-s001.pdf]

Table S1. Consolidated criteria for reporting qualitative studies (COREQ): 32-item checklist.

No. Item	Guide questions/description	Reported on Page #
**Domain 1: Research team and reflexivity**		
Personal Characteristics		
1. Interviewer/facilitator	Which author/s conducted the interview or focus group?	4
2. Credentials	What were the researcher's credentials? e.g., PhD, MD	UM: PhD in Health Service Research<br>TTK: PhD in Sociology
3. Occupation	What was their occupation at the time of the study?	Both: researcher
4. Gender	Was the researcher male or female?	One female and one male
5. Experience and training	What experience or training did the researcher have?	One of the researchers had prior qualitative and quantitative research experience. The other had prior experience in qualitative and quantitative document analysis.
Relationship with participants		
6. Relationship established	Was a relationship established prior to study commencement?	3–4
7. Participant knowledge of the interviewer	What did the participants know about the researcher? e.g., personal goals, reasons for doing the research	4
8. Interviewer characteristics	What characteristics were reported about the interviewer/facilitator? e.g., Bias, assumptions, reasons and interests in the research topic	12
**Domain 2: study design**		
Theoretical framework		
9. Methodological orientation and Theory	What methodological orientation was stated to underpin the study? e.g., grounded theory, discourse analysis, ethnography, phenomenology, content analysis	4
Participant selection		
10. Sampling	How were participants selected? e.g., purposive, convenience, consecutive, snowball	3-4
11. Method of approach	How were participants approached? e.g., face-to-face, telephone, mail, email	4
12. Sample size	How many participants were in the study?	4
13. Non-participation	How many people refused to participate or dropped out? Reasons?	4
Setting		
14. Setting of data collection	Where was the data collected? e.g., home, clinic, workplace	4
15. Presence of non-participants	Was anyone else present besides the participants and researchers?	4
16. Description of sample	What are the important characteristics of the sample? e.g., demographic data, date	4
Data collection		
17. Interview guide	Were questions, prompts, guides provided by the authors? Was it pilot-tested?	3
18. Repeat interviews	Were repeat interviews carried out? If yes, how many?	no
19. Audio/visual recording	Did the research use audio or visual recording to collect the data?	4
20. Field notes	Were field notes made during and/or after the interview or focus group?	no
21. Duration	What was the duration of the interviews or focus group?	4
22. Data saturation	Was data saturation discussed?	3
23. Transcripts returned	Were transcripts returned to participants for comment and/or correction?	no
**Domain 3: analysis and findings**		
Data analysis		
24. Number of data coders	How many data coders coded the data?	4
25. Description of the coding tree	Did authors provide a description of the coding tree?	4
26. Derivation of themes	Were themes identified in advance or derived from the data?	4
27. Software	What software, if applicable, was used to manage the data?	4
28. Participant checking	Did participants provide feedback on the findings?	no
Reporting		

29. Quotations presented	Were participant quotations presented to illustrate the themes/findings? Was each quotation identified? e.g., participant number	5–8
30. Data and findings consistent	Was there consistency between the data presented and the findings?	5
31. Clarity of major themes	Were major themes clearly presented in the findings?	5–8
32. Clarity of minor themes	Is there a description of diverse cases or discussion of minor themes?	10

Supplementary File 1

Definition of important terms

eHealth: the use of information and communication technologies (ICT) for health

E-messages: is an electronic information exchange solution between the local EHR-systems in primary and/or specialist health care.

Discharge summary: the main source of standardised clinical information between health care services and a vital information source for the health professionals involved in a patient's treatment and care

Intermediate departments (IMD): IMD are organised through the municipal health services in Norway. Intermediate means between the specialist (hospitals) and municipal health service. Patients admitted to IMD require more advanced treatment than the municipal health services are able to offer before returning to their own home or home care services; i.e. medical treatment or physical rehabilitation

Summary Care Record (SCR): an online service containing important and critical information about citizens' health. The SCR contains critical information, a pharmaceutical summary, appointment history (hospitals), patient data (relative, GP) and communication challenges. https://helsenorge.no/kjernejournal/kjernejournal-for-safer-healthcare

Shared Medication list (SML): The SML will be an overview showing current medications and drug reactions for a patient. The doctor is responsible for creating and updating the SML. The SML will be uploaded through the Norwegian Prescription Mediator for doctors, and through the SCR for other health professionals involved in the patient´s treatment. As the SML is being prepared, the role and access of pharmacists has not yet been clarified.
